# Drug Resistance to Anti-Tuberculosis Drugs: A Cross-Sectional Study From Makkah, Saudi Arabia

**DOI:** 10.7759/cureus.17069

**Published:** 2021-08-10

**Authors:** Ahmad M Al-Hayani, Shady A Kamel, Sami S Almudarra, Majed Alhayani, Ahmed Abu-Zaid

**Affiliations:** 1 Saudi Field Epidemiology Training Program, Ministry of Health, Riyadh, SAU; 2 College of Medicine, King Abdulaziz University, Jeddah, SAU; 3 College of Graduate Health Sciences, The University of Tennessee Health Science Center, Memphis, USA

**Keywords:** tuberculosis, multidrug-resistant tuberculosis, polydrug-resistant tuberculosis, makkah, saudi arabia, isoniazid, rifampicin

## Abstract

Aim

The aim of this study was to investigate the patterns and determinants of drug resistance to tuberculosis (TB) in a large population from Makkah, Saudi Arabia.

Methods

A retrospective, cross-sectional cohort study was conducted on all patients with TB who were referred to the National Tuberculosis Prevention Program in Makkah, Saudi Arabia, between January 2016 and September 2020. For each TB case, demographic data were collected in addition to the results of drug susceptibility testing (DST) for anti-TB drugs. The Statistical Package for Social Sciences (SPSS) software for Windows, version 23 (IBM Corporation, Armonk, NY, USA), was used for the statistical analysis.

Results

A total of 472 TB-confirmed cases were included in the analysis. The mean ± standard deviation of the age was 38.5 ± 17.7 years. The vast majority of patients were male (62.7%) and had pulmonary TB (91.7%). Only a small proportion of the patients with TB had diabetes mellitus (8.5%). Overall, the prevalence of monodrug-resistant TB ranged from 2.1% to 3.4%. Specifically, the prevalence of monodrug-resistant TB to isoniazid and streptomycin was ranked first and was equal to 3.4%. Pyrazinamide had the lowest prevalence of monodrug-resistant TB (2.1%). The prevalence of polydrug-resistant TB (PDR-TB) and multidrug-resistant TB (MDR-TB) was 1.5%. In the univariate analysis, sex (male) was the only sociodemographic factor that significantly correlated with a higher prevalence of MDR-TB.

Conclusions

This is the second study from Makkah to analyze the prevalence and associated risk factors of MDR-TB among patients from Makkah. Our data demonstrated that the prevalence of monodrug-resistant TB and MDR-TB was low (2.1%-3.4% and 1.5%, respectively). Diabetes mellitus was not a substantial factor correlated with a higher occurrence of MDR-TB. Additional epidemiologic studies are required to validate our results.

## Introduction

Tuberculosis (TB) continues to be a chief healthcare predicament worldwide and a prominent cause of mortality from a solitary infectious disease agent [1]. More than 10 million new cases of TB and 1.5 million mortalities were documented in 2018 [2]. By the end of 2035, the World Health Organization (WHO) aspires to accomplish a dramatic decline in the frequency of TB and its mortality by 90% and 95%, respectively [3].

Although TB can be successfully cured in the vast majority of patients, some individuals develop resistance to first-line anti-TB therapy, comprising isoniazid, rifampicin, pyrazinamide, ethambutol, and streptomycin [4]. Polydrug-resistant TB (PDR-TB) occurs when a patient develops resistance to two or more first-line anti-TB agents other than isoniazid and rifampicin [5]. On the other hand, multidrug-resistant TB (MDR-TB) occurs when a patient develops resistance to both isoniazid and rifampicin with or without other first-line agents [6]. Approximately half a million TB cases are diagnosed with MDR-TB [2]. Unfortunately, MDR-TB is associated with costly treatment expenses, lower rates of treatment success, and higher burdens of morbidity and mortality [7-9].

TB remains a major public health issue in Saudi Arabia. According to the most recent statistics, the yearly TB incidence is 10 per 100,000 individuals [2]. A large national study from Saudi Arabia demonstrated that MDR-TB rates among new and previously treated cases were 1.8% and 15.9%, respectively [10]. Other national registry data from the Ministry of Health stated that the prevalence rate of MDR-TB was 4.4% [11].

The occurrence rate of MDR-TB varies according to the region/city of the report [12], and these geographic differences need to be recognized. Makkah, a city in the Western province of Saudi Arabia, serves as the principal hub for two mass religious gatherings, namely the Hajj and Umrah pilgrimages. While Hajj pilgrimage takes place annually by the end of the Hijri calendar, Umrah pilgrimage takes place throughout the year. These two pilgrimages attract millions of individuals worldwide, including Asian and African countries with a high TB burden [1]. Thus, it is critical to explore the prevalence and patterns of MDR-TB in this vulnerable city. So far, only one study has aimed to explore the prevalence and determinants of MDR-TB in Makkah [13]. This study was conducted at the Al-Noor Specialist Hospital and evaluated the records of only 158 patients with TB-confirmed cases. Hence, additional large-sized data are warranted to provide more comprehensive and high-quality evidence.

This study aimed to investigate the patterns and determinants of drug resistance to TB among a large sample from Makkah, Saudi Arabia.

## Materials and methods

The study protocol was approved by the Institutional Review Board of King Abdulaziz City for Science and Technology, Riyadh, Saudi Arabia. A retrospective, cross-sectional cohort study was conducted on all TB-confirmed patients, irrespective of age, who were referred to the National Tuberculosis and Prevention Program in Makkah, Saudi Arabia, between January 2016 and September 2020.

The TB diagnosis was confirmed by various methods, including chest radiography, sputum culture, microscopy, or molecular assays. TB resistance was established using drug susceptibility testing (DST). To isolate and identify the mycobacterium agent, Ziehl-Neelsen (ZN) staining was performed to examine acid-fast bacilli. Culture and DST were performed using the mycobacteria growth indicator tube (MGIT) medium and BACTEC MGIT 960 instrument (Becton, Dickinson and Company, Maryland, United States of America), as reported previously [14]. The final drug concentrations utilized for DST included 5 μg/ml for ethambutol, 0.1 μg/mL for streptomycin, 1 μg/mL for rifampicin, 0.1 μg/mL for isoniazid, and 25 μg/mL for pyrazinamide. For selected samples, the GenXpert Mycobacterium tuberculosis complex/resistance to rifampicin (MTB/RIF) assay (Cepheid, California, United States of America) was used to screen for the Mycobacterium tuberculosis complex and resistance to rifampicin.

Demographic data were collected for each TB case (i.e., age, sex, nationality, disease site, diabetes mellitus, renal failure, acquired immunodeficiency syndrome, immunosuppression, cancer, and Bacillus Calmette-Guérin vaccination) in addition to the results of the DST for TB. DST results focused on identifying the rates of monodrug-resistant TB (resistance to one anti-TB drug), PDR-TB (resistance to two or more anti-TB drugs other than isoniazid and rifampicin), and MDR-TB (resistance to at least both isoniazid and rifampicin with or without resistance to other drugs).

The Statistical Package for Social Sciences (SPSS) software for Windows, version 23, was used for statistical analysis (IBM Corporation, Armonk, NY, USA). Descriptive data were presented as numbers and percentages or means and standard deviations, as appropriate. The Chi-squared test was used to examine correlations between select patient demographics and the occurrence of MDR-TB. 

## Results

A total of 472 records of TB-confirmed cases were included in the analysis. Table 1 summarizes the sociodemographic characteristics of the patients. The mean ± standard deviation of age was 38.5 ± 17.7 years (range: 2-101 years). The prevalence of TB was equally divided between Saudi and non-Saudi nationals (50.4% and 49.6%, respectively). Most patients were male (62.7%) and had pulmonary TB (91.7%). Only a small proportion of TB patients had diabetes mellitus (8.5%), renal failure (23%), acquired immunodeficiency syndrome (3%), immunosuppression (1.9%), and cancer (1.5%). Equally, 5.7% of patients had a previous history of TB and received the Bacillus Calmette-Guérin vaccine.

**Table 1:** The sociodemographics of patients.

Sociodemographic	n (%)
Nationality	
Saudi	238 (50.4)
Non-Saudi	234 (49.6)
Sex	
Male	296 (62.7)
Female	176 (37.7)
Type	
Pulmonary	433 (91.7)
Extra-pulmonary	36 (7.6)
Both	3 (0.6)
Diabetes mellitus	
Yes	40 (8.5)
No	432 (91.5)
Renal failure	
Yes	11 (2.3)
No	461 (97.7)
Acquired immunodeficiency syndrome	
Yes	14 (3)
No	458 (97)
Immunosuppression	
Yes	9 (1.9)
No	463 (98.1)
Cancer	
Yes	7 (1.5)
No	465 (98.5)
Bacillus Calmette–Guérin vaccine	
Yes	27 (5.7)
No	445 (94.3)
Previous history of tuberculosis	
Yes	27 (5.7)
No	445 (94.3)

Table 2 outlines the patterns of drug resistance to anti-TB drugs. Overall, the prevalence of monodrug-resistant TB ranged from 2.1% to 3.4%. Specifically, the prevalence of monodrug-resistant TB to isoniazid and streptomycin was ranked first and was equal to 3.4%. Pyrazinamide had the lowest prevalence of monodrug-resistant TB (2.1%). The prevalence of PDR-TB and MDR-TB was 1.5%.

**Table 2:** Patterns of resistance to anti-tuberculosis drugs.

Type of tuberculosis resistance	n (%)
Monodrug-resistant tuberculosis	
Isoniazid	16 (3.4)
Pyrazinamide	10 (2.1)
Ethambutol	12 (2.5)
Rifampicin	13 (2.8)
Streptomycin	16 (3.4)
Polydrug-resistant tuberculosis	
Pyrazinamide plus ethambutol	5 (1.1)
Pyrazinamide plus streptomycin	7 (1.5)
Ethambutol plus streptomycin	7 (1.5)
Multidrug-resistant tuberculosis	7 (1.5)

Table 3 depicts the univariate analysis between the patient sociodemographics and the prevalence of MDR-TB. Sex (male) was the only sociodemographic factor that significantly correlated with a higher prevalence of MDR-TB (all the seven cases of MDR-TB occurred in male individuals). 

**Table 3:** Univariate analysis (two-tailed chi-squared test) between patient sociodemographics and prevalence of multidrug-resistant tuberculosis. Statistical significance was determined as p-value <0.05.

Sociodemographic variable	p-value
Nationality	0.244
Sex	0.04
Tuberculosis type	0.726
Diabetes mellitus	0.541
Renal failure	0.681
Acquired immunodeficiency syndrome	0.641
Immunosuppression	0.710
Cancer	0.744
Bacillus Calmette–Guérin vaccine	0.511
Previous history of tuberculosis	0.511

## Discussion

This is one of the limited studies from the Western province of Saudi Arabia to scrutinize the prevalence and associated risk factors of MDR-TB among patients from Makkah. Our study revealed that the prevalence of monodrug-resistant TB ranged from 2.1% to 3.4%. Conversely, the prevalence rates for PDR-TB and MDR-TB were 1.5%. Additionally, diabetes and a previous history of TB were not substantial factors correlated with a higher occurrence of MDR-TB.

In our study, the distribution of TB cases was roughly divided between Saudi and non-Saudi nationals. This finding suggests that expatriates contribute substantially to the overall TB prevalence in Makkah, which harbors two large-sized religious gatherings and welcomes millions of people from all over the world.

Our data revealed that the prevalence rate of MDR-TB among TB-confirmed patients from Makkah was low (1.5%). Our reported figure differed from two national registry data reporting MDR-TB prevalence rates of 15.9% [10] and 4.4% [11]. Indeed, MDR-TB remains an ongoing encumbrance and several national reports from Saudi Arabia have revealed that MDR-TB rates vary substantially in accordance with the reporting region/city [12]. For example, it has been documented that the rate of MDR-TB was 1.4% in the Eastern provinces [12], 20.6% in Najran [15], 2.5-6.7% in Riyadh [16,17], 4% in Al-Madinah Al-Munawarah [18], and 20% in Jeddah [19].

With regard to Makkah, only one study thus far has endeavored to examine the prevalence and determinants of MDR-TB [13]. This study was a single-center experience from the Al-Noor Specialist Hospital, reporting only a total of 158 patients. This study revealed that the prevalence rate of MDR-TB was 5% [13]. Moreover, this study demonstrated that age, lung disease, and a previous history of TB were substantial factors correlated with MDR-TB [13]. Our present study is characterized by a larger sample size and a more holistic representation of Makkah population, as data were derived from the registry of the Central TB Laboratory in Makkah.

Isoniazid and streptomycin were the two most frequent monodrug-resistant anti-TB agents (3.4%). Our data mirrored the findings of another study conducted in Makkah by Sambas et al. [13] and other studies conducted in Jeddah [19], Riyadh [20], and Dhahran [21]. Moreover, our data were consistent with a comparable pattern previously chronicled by two national registries in Saudi Arabia [10,11]. Indeed, isoniazid is the most commonly effective yet most frequently resistant anti-TB agent worldwide [22].

Sambas et al. [13] revealed that age, lung disease, and a previous history of TB were substantial factors correlated with MDR-TB among patients from Makkah. However, these observations did not occur in our study. The lack of statistically significant differences in our study between the abovementioned risk factors and hazard of MDR-TB could be ascribed to the small number of MDR-TB cases (1.5%). The correlation between age and the hazard of MDR-TB is inconsistent across the published literature. While some studies revealed a positive relationship between advanced age and risk of MDR-TB [13,23], other studies from Asia [11] and Europe [24] found a negative correlation. On the other hand, several studies have identified no significant connection between age and risk of MDR-TB [25-27]. Earlier studies depicted a positive link between coexisting chronic diseases, such as diabetes mellitus and the risk of MDR-TB [28]. In addition, a prior history of TB has been extensively recognized as a substantial contributing factor to MDR-TB [10,11]. Our study revealed that male sex was positively correlated with a higher rate of MDR-TB, consistent with earlier systematic reviews [24,29].

It is critical to gauge the extent of resistance to TB, particularly the prevalence rates of monodrug-resistant TB and MDR-TB. This is because resistance to anti-TB drugs is disadvantageously linked to limited therapeutic alternatives. Also, resistance to anti-TB drugs demands the utility of several costly drugs which are associated with high toxicities. Moreover, MDR-TB is unfavorably associated with higher morbidity and mortality compared to drug-susceptible TB. Lastly, MDR-TB is inconveniently correlated with higher healthcare costs [7,30]. Epidemiologic studies on anti-TB drug resistance are crucial to healthcare policymakers and stakeholders. Such studies permit the development of proper surveillance initiatives to ensure effective control and prevention of MDR-TB, along with training the treating physicians about the recommended initial anti-TB regimens for a particular geographic region.

Our study has several strengths. It is the second-ever study about the prevalence of MDR-TB in Makkah; however, with a larger sample size than that reported by Sambas et al. [13]. Our data also included only TB-confirmed cases extracted from the Central TB Laboratory in Makkah, highlighting a broader representation of the population of Makkah. Nevertheless, our study has some limitations. Such limitations comprise the retrospective and cross-sectional study design, which does not reliably permit instituting a temporal correlation between the exposure and outcome variables. Also, due to the small number of MDR-TB cases, it was not possible to confidently identify relationships between risk of MDR-TB and some clinical and demographic parameters.

## Conclusions

This is the second-ever study from the Western province of Saudi Arabia to analyze the prevalence and associated risk factors of MDR-TB among patients from Makkah. Our data demonstrated that the prevalence rates of monodrug-resistant TB and MDR-TB were low (2.1%-3.4% and 1.5%, respectively). Diabetes and a previous history of TB were not substantial factors correlated with a higher occurrence of MDR-TB. Additional epidemiologic studies are required to validate our results.
